# Malaria morbidity in Papua Indonesia, an area with multidrug resistant *Plasmodium vivax *and *Plasmodium falciparum*

**DOI:** 10.1186/1475-2875-7-148

**Published:** 2008-08-02

**Authors:** Muhammad Karyana, Lenny Burdarm, Shunmay Yeung, Enny Kenangalem, Noah Wariker, Rilia Maristela, Ketut Gde Umana, Ram Vemuri, Maurits J Okoseray, Pasi M Penttinen, Peter Ebsworth, Paulus Sugiarto, Nicholas M Anstey, Emiliana Tjitra, Richard N Price

**Affiliations:** 1 National Institute of Health Research and Development, Ministry of Health, Jakarta, Indonesia; 2 District Health Authority, Timika, Papua, Indonesia; 3 Mahidol-Oxford Tropical Medicine Research Unit Faculty of Tropical Medicine Mahidol University, Bangkok, Thailand; 4 Menzies School of Health Research-National Institute of Health Research and Development Malaria Research Program, Timika, Indonesia; 5 Public Health Malaria Control, International SOS, Tembagapura, Papua, Indonesia; 6 Charles Darwin University, Darwin, NT, Australia; 7 Mitra Masyarakat Hospital, Timika, Indonesia; 8 International Health Division, Menzies School of Health Research, Darwin, Australia; 9 Centre for Vaccinology & Tropical Medicine, Nuffield Department of Clinical Medicine, Churchill Hospital, Oxford, UK

## Abstract

**Background:**

Multidrug resistance has emerged to both *Plasmodium vivax *and *Plasmodium falciparum *and yet the comparative epidemiology of these infections is poorly defined.

**Methods:**

All laboratory-confirmed episodes of malaria in Timika, Papua, Indonesia, presenting to community primary care clinics and an inpatient facility were reviewed over a two-year period. In addition information was gathered from a house-to-house survey to quantify the prevalence of malaria and treatment-seeking behaviour of people with fever.

**Results:**

Between January 2004 and December 2005, 99,158 laboratory-confirmed episodes of malaria were reported, of which 58% (57,938) were attributable to *P. falciparum *and 37% (36,471) to *P. vivax*. Malaria was most likely to be attributable to pure *P. vivax *in children under one year of age (55% 2,684/4,889). In the household survey, the prevalence of asexual parasitaemia was 7.5% (290/3,890) for *P. falciparum *and 6.4% (248/3,890) for *P. vivax*. The prevalence of *P. falciparum *infection peaked in young adults aged 15–25 years (9.8% 69/707), compared to *P. vivax *infection which peaked in children aged 1 to 4 years (9.5% 61/642). Overall 35% (1,813/5,255) of people questioned reported a febrile episode in the preceding month. Of the 60% of people who were estimated to have had malaria, only 39% would have been detected by the surveillance network. The overall incidence of malaria was therefore estimated as 876 per 1,000 per year (Range: 711–906).

**Conclusion:**

In this region of multidrug-resistant *P. vivax *and *P. falciparum*, both species are associated with substantial morbidity, but with significant differences in the age-related risk of infection.

## Background

The true burden of malaria outside of sub-Saharan Africa has been underestimated [1], largely due to the lack of accurate estimates from India and Indonesia, whose combined population accounts for 20% of the world's population. In Indonesia, almost half of the country's population of 250 million live in malaria-endemic areas. In Java and Bali, where approximately 70% of the country's population live, malaria is hypoendemic and vivax malaria predominates. In the outer island groups, the incidence of malaria is much higher with the prevalence of *Plasmodium falciparum *and *Plasmodium vivax *infection almost equal. According to the National Health Household Survey in 2001, approximately 15 million people with clinical malaria sought treatment [2], three million of whom attend government health centres and hospitals. However, the true incidence of malaria is unknown since laboratory-confirmation is rare and only 20% of patients with symptomatic malaria seek treatment at government health facilities [3].

National statistics suggest an increase in malaria in Indonesia over the last three years. Although this is a consequence of several factors, the emergence and spread of antimalarial drug resistance is likely to be a key determinant. South-east Asia has been the focus for emerging drug resistant strains of *P. falciparum *for more than 40 years, but drug resistance in *P. vivax *has been slower to evolve. The first chloroquine resistant isolates of *P. vivax *were reported from Papua, Indonesia and Papua New Guinea in 1989 [4,5]. These regions now have the highest rates of drug-resistant *P. vivax *in the world [6]. A clinical drug study conducted in southern Papua in 2004, highlighted a risk of failure within 28 days of 65% after chloroquine monotherapy for *P. vivax *and 48% after chloroquine plus sulphadoxine-pyrimethamine for *P. falciparum *[7]. The risk of of treatment failure elsewhere in Papua has reached 95% [8]. Recent studies from both the north and south of Papua, have highlighted that multidrug-resistant P. vivax in this region is associated with severe malaria and even death [9,10]. As a consequence of the high levels of antimalarial drug resistance, first-line treatment of uncomplicated malaria, for any species of infection, was changed to dihydroartemisinin-piperaquine in March 2006.

The aim of the present study was to document further the epidemiology of malaria in this region prior to the introduction of an artemisinin combination therapy, as part of an ongoing analysis of the impact of policy change. To achieve this a comprehensive epidemiological study was conducted using surveillance data from primary and secondary health facilities and a cross-sectional malaria prevalence survey. At the same time a treatment-seeking behaviour study was also conducted to estimate the burden of malaria occurring outside this surveillance system.

## Methods

### Study site and population

**Figure 1:**
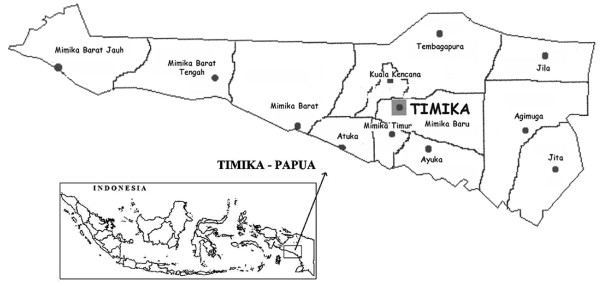
Location of study area in Timika, southern Papua, Indonesia.

The Mimika district lies on the southern coast of Papua in Eastern Indonesia (Figure 1), covering an area of 21,522 square-kilometres with 12 sub-districts and 85 villages. The area is largely forested with both coastal and mountainous areas. Each year a total of approximately 5.5 metres of rainfall is recorded with peaks in July to September and December (unpublished data). At the last census in 2004, the population in the lowlands was reported as 130,000. One hospital, the Rumah Sakit Mitra Masyarakat (RSMM) in the town of Timika, services the whole district and is the only hospital available for the lowland population. Due to the presence of a local mine, there is economic migration, with the local population increasing by an estimated 16% per year. This has resulted in the diverse ethnic origin of the local population, with highland Papuans, lowland Papuans and non-Papuans all resident in the region. Healthcare for the population is provided by the public clinics of the local ministry of health, the Public Health Malaria Control programme (PHMC) of the mine, the RSMM hospital and the private sector.

Malaria transmission is perennial, but restricted to the lowland area where it is associated with three mosquito vectors: *Anopheles koliensis*, *Anopheles farauti *and *Anopheles punctulatus *[11,12]. Entomological inoculation rates vary between 1 and 4 infected bites per year (unpublished data). Bed net coverage is estimated to be approximately 40%. In view of the high number of infections in non-immune patients, local protocols recommend that all patients with patent parasitaemia at any level are given antimalarial therapy. At the time of the study local treatment guidelines advocated chloroquine plus sulphadoxine-pyrimethamine for *P. falciparum *and chloroquine monotherapy for non-falciparum malaria. An assessment of local treatment regimens in 2004 highlighted that the day-28 cure rate of chloroquine monotherapy was less than 35% for patients with *P. vivax *and that of chloroquine plus sulphadoxine-pyrimethamine was only 52% for patients with *P. falciparum *[7]. Local and national guidelines also recommend that patients with *P. vivax *parasitaemia over 1 years old, receive 14 days unsupervised treatment with primaquine, however adherence to and effectiveness of this regimen in this setting is not known.

### Study design

Data was collected from routine health facilities surveillance data and from a cross-sectional survey.

### Routine surveillance data

Data from health facilities were collated to determine the number of patients treated for laboratory-confirmed malaria between January 2004 and December 2005. The established routine malariometric surveillance network comprised 14 primary health clinics and the RSMM hospital. Four primary health clinics were funded by the local ministry of health and ten were funded by the public health malaria control (PHMC) programme of the local mine. The total number of malaria cases reported were equally divided between the hospital, the PHMC clinics and the local ministry of health clinics.

At all of these facilities malaria treatment guidelines require that treatment should be preceded by microscopic confirmation of thick or thin blood film. Parasite species was assessed by surveillance facility microscopists from Giemsa-stained thick blood films and peripheral parasitaemia determined from the number of parasites per 200 white blood cells and quantified as 1+, 2+, 3+ or 4+. A thick smear was considered negative on initial review if no parasites were seen in 100 high power fields. A thin smear was also examined to confirm parasite species and used for quantification if parasitaemia was greater than 200 per 200 WBC. All laboratories in the surveillance network provide weekly aggregated data on the number of slides read, and the species reported according to four age categories (less than 1 year, 1 to 4 years, 5 to 9 years and 10 or more years). The surveillance microscopists participate in ongoing quality assurance, training and monitoring. In 2004 the microscopy service at the hospital was assessed for accuracy, and a random sample of 1200 positive slides was selected for rereading by an independent microscopist with more than 10 years' experience. In total, slides were available in 97% (1,158/1,200) cases with concordance between the readings of 89% (1,032/1,158). Of the 126 discordant slides 18 (14%) were negative on second rereading, 46 (37%) monoinfections were reread as mixed species, 47 (37%) mixed infections were reread as monoinfections. A further 6 slides with *P. falciparum *infections were reread as *P. vivax*, and 9 slides with *P. vivax *infections were reread as *P. falciparum*.

In the hospital, data were collected from individual patient records. For those attending the hospital outpatient clinic, information of the hospital number, date of visit, age and sex of the patients and diagnosis, including the species of infection if malaria, were registered on computerized hospital records (Q-Pro_; Jakarta, Indonesia). All patients admitted to hospital for more than 12 hours were reviewed by a member of the onsite research team, and details of their visit documented on a standard report form which included, for this study, the hospital record number, data of admission, age and sex of the patient [9].

### Cross-sectional household survey

A cross-sectional survey, was conducted during July to December 2005, to determine the demographic breakdown of the local population, the population prevalence of malaria and anaemia, and document the treatment-seeking behaviour and costs incurred by febrile illness. The survey methodology and results will be described in more detail separately. For the treatment seeking survey, the sample size required to determine the true prevalence of households with someone with a recent history of fever was based on an estimated prevalence of 40% and population size of 140,000; a multiplication factor of 2 was used to take into account the design effect due to cluster sampling. A sample size of 800 households was required to achieve an estimate of prevalence with greater than 95% confidence [13].

Households were chosen by three-stage cluster random sampling, according to WHO guidelines [14]. Household members were defined as anyone who lived under one roof, ate from one kitchen and had resided in the study area for at least six months. For each member, demographic information and fever history were recorded using a standardized questionnaire. When the member was present, weight and height were documented and a finger prick of blood taken for blood film examination. Axillary temperature was recorded and respondents defined as symptomatic if they reported history of fever in the last 24 hours or had an axillary temperature of 37.5°C or above. All household members who reported fever in the last months were asked to complete a questionnaire on treatment seeking behaviour.

### Blood film examination

Blood film examination was carried out for all the facility surveys. The peripheral parasitaemia was calculated assuming a white cell count of 7,300 μl-1 ^**1 **^[7]. All positive blood films and 10% negative blood films were rechecked in the National Institute of Health Research and Development reference laboratory in Jakarta, and results that differed from those obtained in the unit laboratory were reviewed by the two head laboratory technologists. Microscopically confirmed malaria cases were treated according to Indonesia Ministry of Health guidelines. Children and adults found to be anaemic either on clinical examination or after determination of haemoglobin levels were provided iron supplementation according to local guidelines. Other common ailments detected clinically were also treated.

### Statistical analysis

Data on patients diagnosed with malaria were single entered into Epi Info 6 (U.S. Centers for Disease Control and Prevention, Atlanta, Georgia, USA) and data from the household surveys double entered and validated using EpiData 3.02 software (EpiData Asoociation, Odense, Denmark). Analysis was performed using SPSS for Windows (vs 15 SPSS Inc, Chicago, Illinois, USA) and STATA statistical software (Version 8, Stata Corp. TX). The Mann-Whitney U test or Kruskal-Wallis method were used for nonparametric comparisons, and Student's t-test or one-way analysis of variance for parametric comparisons. For categorical variables, percentages and corresponding 95% confidence intervals (95% CI) were calculated using Wilson's method. Proportions were examined using χ^2 ^with Yates' correction or by Fisher's exact test. Pyrogenic thresholds were defined using the receiver operator curve, and Youlden's Index calculated as sensitivity plus specificity minus 1.

In order to estimate the annual population incidence of malaria, data from the fever treatment seeking behaviour study were used to estimate the proportion of patients with malaria attending clinics participating in the surveillance network. In case the treatment seeking behaviour of patients with non-malarial fever differed significantly from those with malaria, we used an algorithm to assign each respondent a malaria diagnosis. This was done according to whether malaria was confirmed by a blood test, the treatment people received, and the status of the blood film at the time of the household survey. A sensitivity analysis was performed by assuming best-case and worse case scenarios where there was uncertainty in the algorithm.

### Ethical considerations

The study was approved by the Ethics committee of the National Institute of Health Research and Development, Indonesian Ministry of Health (Jakarta, Indonesia) and the Ethics committee of Menzies School of Health Research (Darwin, Australia). Written informed consent was obtained from adult patients and parents of enrolled children.

## Results

### Health facilities data

**Figure 2:**
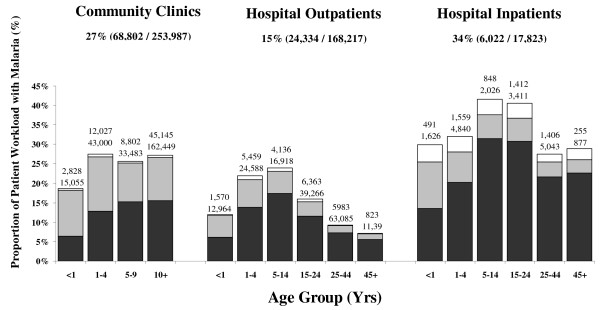
Age-stratified proportions of total patient workload diagnosed with pure *P. falciparum *(dark bars), pure *P. vivax *(light bars) and mixed falciparum and vivax infections (white bars) at each type of healthcare facility. Footnote: For each age group the lower number above the column is the total number of patients reviewed (= microscopy examinations in community clinics, or patients seen or admitted at the hospital) and the upper number represents the total number of patients with malaria.

Between January 2004 and December 2005, 99,158 laboratory-confirmed episodes of malaria were reported from patients seeking treatment at health facilities in the surveillance network. Of these, 68,802 (69%) were reported from community outpatients, 24,334 (25%) hospital outpatients and 6,022 (6.1%) from hospital admissions. Laboratory confirmation of malaria was made in 27% (68,802/253,987) of patients who had a malaria smear at community clinics. At the hospital, 15% (24,334/168,217) of the total outpatient workload and 34% (6,022/17,823) of the total inpatient workload was attributable to patients with malaria (Figure 2). Although treatment guidelines recommend that all patients have microscopic confirmation of malaria prior to treatment, a further 2,148 outpatients were reportedly diagnosed and treated following clinical diagnosis, ie 8.1% (2,148/26,482) of all outpatients treated for malaria.

Children under five years of age, accounted for 22% (14,855/68,802) of community outpatients with malaria, 29% (7,029/24,334) of hospital outpatients and 34% (2,050/6,022) of patients admitted to hospital (overall p < 0.001).

In total 58% (57,938) of cases had infection with *P. falciparum *and 37% (36,471) *P. vivax *(Table 1). The proportion of malaria due to *P. vivax *was greatest in community outpatients (42% 29,144/68,802), but fell to 25% (6,181/24,334) in hospital outpatients and 19% (1,146/6,022) in hospital inpatients (p < 0.001). In children under one year of age malaria was most likely to be attributable to infection with pure *P. vivax *(overall 55% 2,684/4,889) and this was apparent in all types of facility; overall p < 0.001 (Figure 2). Malaria attributable to *P. vivax *fell to 43% (8,112/19,045) in children aged 1–4 years, to 33% (4,029/12,096) in children 5 to 9 years old and 34% (21,618/63,077) thereafter.

**Table 1:** Number of laboratory-confirmed malaria cases in 2 years of community and hospital surveillance (January 2004–December 2005)

	**Total number of patients ** **with confirmed malaria**	***P. falciparum***	***P. vivax***	***P. malariae***	**Mixed Infections**
**Community outpatients**	68,802	36,848 (53.6%)	29,144 (42.4%)	1,325 (1.9%)	1,485 (2.2%)
**Hospital outpatients**	24,334	16,895 (69.4%)	6,181 (25.4%)	382 (1.6%)	876 (3.6%)
**Hospital inpatients**	6,022	4,195 (69.7%)	1,146 (19.0%)	68 (1.1%)	613 (10.2%)
**Total**	99,158	57,938 (58.4%)	36,471 (36.8%)	1,775 (1.8%)	2,974 (3.0%)

### Household survey

**Figure 3:**
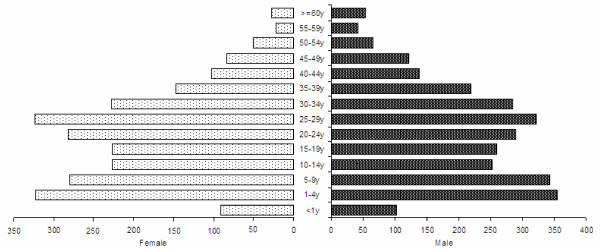
Population by age group and gender in the community household survey.

Demographic data was gathered for 5,255 people from 825 households. Overall 46% (2,409) were female with 6% (92/1,441) of women of childbearing age pregnant. Of the 5,255 respondents questioned 28% (1,494) were highland Papuans, 26% (1,371) lowland Papuans, with the remaining 45% (2,390) of non-Papuan origin. The median age was 21 years [Interquartile Range: 8–32], with 17% (870) children aged less than 5 years (Figure 3). Of the 3,284 adults (age > 15 years), 26% (866) reported being housewives, 14% (456) were private employees, 9% (299) were farmers and 20% (668) were unemployed. At the time of interview, 3,896 household members were present, with microscopy results available in 3,890, representing 74% of all household members. Absent members were most likely to be adults (76%, 1,032/1,359) of whom 78% were male (805/1,032).

**Figure 4:**
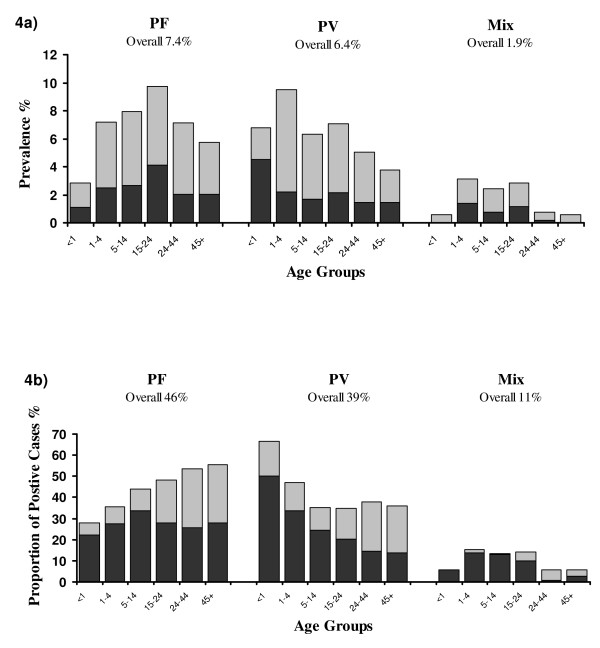
**a) **Age-stratified parasitaemia prevalence rates in the household survey for *P. falciparum, P. vivax *and mixed infections for febrile (dark bars) and afebrile respondents (light bars). **b) **Proportions of all parasitaemic cases attributable to *P. falciparum, P. vivax *and mixed infections for Papuans (dark bars) and Non-Papuans (light bars).

The overall prevalence of asexual parasitaemia was 16.3% (634/3,890) with *P. falciparum *infection present in 290 (46%), *P. vivax *infection in 248 (39%), *P. malariae *in 24 (4%) and mixed infections in 72 (11%) (Figure 4a). The prevalence of malaria varied with age. The median age of respondents with* P. falciparum* parasitaemia was 19 years, with prevalence peaking at 10% (69/707) in young adults aged 15–24 years. In contrast, the median age of those with *P. vivax *infection was significantly lower (13.5 years, p = 0.01) with a peak of 9.5% (61/642) in children aged one to four years (Figure 4a). The proportion of patients with parasitaemia due to *P. vivax *infection was greatest in infancy (67% 12/18) falling to 47% (61/130) in children aged one to four years, with no change thereafter (overall 36% 175/486); p = 0.004 (figure 4b). The overall risk of parasitaemia was 19% (402/2067) in Papuans, significantly higher than in non-Papuans (12.7% 232/1,823); p = 0.001. The relative proportion of parasitaemia attributable to non-Papuans was 22% (66/230) in children under 15 years, but rose to 49% (166/338) in adults; p < 0.001 (Figure 4b).

### Fever and parasite density

In total 68% (434/634) of respondents with asexual parasitaemia were asymptomatic (afebrile with no history of fever in preceding 24 hours). Although this proportion did not differ for *P. falciparum*, *P. vivax*, or mixed infections, asymptomatic carriage was significantly more likely in people with pure *P malariae *infections (91.7% (22/24); Odds Ratio = 5.3 [95%CI 1.2–33]), p = 0.023. Patients with *P. vivax *parasitaemia were significantly less likely to be afebrile if they were less than 1 year of age (33% 4/12) compared to 75% (85/113) of older children and 70% (86/123) of adults (p = 0.004 and 0.02 respectively), although this age difference was not significant for the other species (Figure 4a). The risk of fever did not vary with sex or ethnicity.

The density of asexual parasitaemia of any species was significantly higher if the patients were febrile (Table 2). A *P. falciparum *parasitaemia above 1,734 μl^-1 ^had a 57% (57/100) sensitivity for predicting fever and a positive predictive value of 61% (57/93). The corresponding threshold for *P. vivax *was 310 μl^-1^, with parasitaemia above this level having a sensitivity of 77% (56/73) and associated with fever in 46% (56/122) of cases.

**Table 2:** Prevalence of asexual parasitaemia in the community household survey.

**Species**	**Prevalence % (n/N)**		**Proportion with Fever % (n)**			**Geometric Mean Parasite ** **count per μl^-1 ^[95% CI]**		
				
**Pf**	7.5%	(290/3890)	With Fever	34%	(100)	2,409	[1,535–3,781]	P < 0.001
			Without Fever	66%	(190)	460	[362–583]	

**Pv**	6.4%	(248/3890)	With Fever	29%	(73)	1,074	[680–1,698]	P < 0.001
			Without Fever	71%	(175)	292	[228–374]	

**Pm**	0.6%	(24/3890)	With Fever	8%	(2)	577		P = 0.295
			Without Fever	92%	(22)	279	[190–410]	

**Mixed**	1.9%	(72/3890)	With Fever	35%	(25)	1,812	[999–3,286]	P = 0.09
			Without Fever	65%	(47)	971	[629–1,498]	

Pf: *Plasmodium falciparum*; Pv: *P. vivax*; Pm: *P. malariae*; Mixed: two or more species

### Estimated incidence

In 2005, a review of 13,766 laboratory records revealed that 96.6% of patients with malaria had one blood film for each episode of malaria, 3.25% had two blood films and 0.13% had three films. The total number of cases treated as community outpatients were therefore multiplied by 0.967 to account for multiple sampling. Hence the total number of patients with confirmed malaria reported by the surveillance network during the two-year period was 95,886.

In the household survey, a history of fever within the last month was reported in 35% (1813/5255) of household members, of whom treatment seeking behaviour could be assessed in 1015 (56%). Of these respondents, 60% (607) were estimated to have had malaria (based upon their treatment and subsequent microscopy at the time of the survey) of whom 39% (238/607) would have been diagnosed and thereby picked up by the surveillance network. The sensitivity analysis defined the lower and upper estimates as 37.7% (261/693) and 48% (206/426) respectively. After correcting for multiple sampling at the community clinics, the surveillance network identified a total 47,943 reports of malaria per annum for a population of 140,4000 people, representing 39% of the total burden of malaria. The overall incidence of malaria, therefore, was estimated as 876 per 1,000 per year (Range: 711–906). The corresponding breakdown was 512 (Range: 416–529) for *P. falciparum*, 322 (Range: 262–333) for *P. vivax*, 15.7 (Range: 12.7–16.2) for *P. malariae*, and 26.3 (Range: 21.3–27.2) for mixed infections.

## Discussion

The burden of *P. vivax *malaria from Asia has been under appreciated, despite the region contributing almost 40% of the world's malaria [1]. *Plasmodium falciparum *and *P. vivax *are equally prevalent over much of the continent, however *P. vivax *is usually assumed to be benign and its associated morbidity often overlooked [6,8]. The situation has been further complicated by the emergence of multidrug resistant Plasmodium. Over the last 50 years, Asia has been the epicentre for the evolution and spread of drug resistant isolates of *P. falciparum*. The latter have subsequently spread to almost all regions of the malarious world, undermining control programmes and significantly exacerbating the burden of malaria [15]. Antimalarial drug resistance in *P. vivax *has taken longer to emerge, the first reports of chloroquine resistance coming from the island of New Guinea in 1989 [4]. However, over the ensuing two decades highly chloroquine-resistant *P. vivax *has become increasingly prevalent in Papua province, with reports of declining efficacy also documented across the Indonesian archipelago as well as in Myanmar, India and South America [6,16-18]. Despite this threat, few studies have addressed the associated epidemiology of malaria in areas where resistance has emerged to both *P. vivax *and *P. falciparum*.

The study presented reviews the epidemiology of malaria in patients attending community clinics and the only referral hospital in southern Papua. In this region cure rates following chloroquine plus sulphadoxine-pyrimethamine have fallen below 50% for both *P. falciparum *and *P. vivax*, with high grade resistance a significant problem [7]. The results demonstrate that malaria accounts for a considerable proportion of the total clinic workload amounting to 15–27% of patients attending outpatient clinics with fever and 34% of all hospital admissions. *P. vivax *was responsible for 42% of malaria treated in the outpatients and 19% of patients admitted to hospital with malaria. In the household surveys, the overall prevalence of malaria was 16%, lower than that reported in West Sumba, Indonesia (31%) [19] and PNG (51%) [20], although the proportion of carriage attributable to *P. vivax *was higher: 39% in Timika compared to 32% in West Sumba and 19% in PNG.

There were significant differences in the age-stratified rates of infection (Figure 2). The workload due to malaria peaked in the 5–14 age group, reaching 24% in hospital outpatients and 42% in inpatients, however this was mainly attributable to an increasing prevalence of clinical infections with *P. falciparum*. In contrast, the burden and proportion of *P. vivax *peaked in early childhood with more than half of malaria in infants attributable to *P. vivax *irrespective of healthcare facility. These findings concur with studies from PNG, Vanuatu and Thailand [20-22]. A notable feature of *P. vivax *infection is the presence of the hypnozoite stage which in equatorial regions can result in frequent relapse and recurrent symptomatic infection. The predominance of *P. vivax *in infancy presumably reflects early exposure to infection, high rates of subsequent recurrence and the rapid acquisition of immunity. The additional impact of chloroquine resistance is difficult to gauge, but likely to further exacerbate the recurrence of malaria following partially effective treatment. In contrast to the PNG study, in which children had acquired almost complete immunity by the age of 9 years old [20], both the present study and a study from Thailand observed a significant number of older children and adults presenting with symptomatic *P. vivax *infections [22]. In Papua, economic immigration has resulted in a high proportion of the population being non-immune, either Papuans from malaria-free highland areas or non-Papuans from provinces with low endemicity. With little or no prior exposure to malaria these migrants are vulnerable to symptomatic infection into adult life. Indeed the proportion of malaria carriage attributable to non-Papuans rose with increasing age (Figure 4b), reflecting not only their vulnerability to symptomatic infection, but also their higher exposure to infection.

In the prevalence survey, approximately a third of patients with peripheral parasitaemia reported an associated fever. Although there was no difference in this proportion between *P. falciparum *and *P. vivax *infections, the pyrogenic thresholds differed considerably: 310 μl^-1 ^for *P. vivax *and 1,734 μl^-1 ^for *P. falciparum*. The ability of *P. vivax *to induce fever at lower levels of parasitemia than *P. falciparum *is well described [22,23] and is consistent with a greater host inflammatory response during infections with *P. vivax*, as evidenced by higher plasma concentration of fever-inducing cytokines, such as TNF, in vivax malaria compared to *P. falciparum *infections with similar parasitaemia [24,25]. Whereas the proportion of symptomatic *P. falciparum *carriage did not change with age, there was a significant reduction in symptomatic carriage with *P. vivax*, falling from 67% in infants to 28% in adults (Figure 4a). All patients with peripheral parasitaemia were treated and, therefore, the proportion of asymptomatic patients who would have become febrile if left untreated can not be gauged.

Since the surveillance system did not include a prospective cohort an estimate of the incidence of clinical infection was derived from the clinic workload and a treatment seeking behaviour survey. The latter will be reported more fully in a separate paper, however the results highlight that only 39% with symptomatic malaria would have sought treatment at a facility in the surveillance network, the majority either staying at home or seeking treatment through the private sector or pharmacies. Extrapolating the total number of reported malaria cases we estimated an incidence of malaria of 512 and 322 per 1,000 population per year for *P. falciparum *and *P. vivax*, respectively.

## Conclusion

In conclusion, in Papua Indonesia both *P. falciparum *and *P. vivax *are associated with significant morbidity. *P. vivax *infection predominated in infancy with symptomatic infections reducing with age, however even in adults this species is responsible for a third of malaria treatments. The presence of high grade chloroquine resistance in both major Plasmodium species in this region, is likely to exacerbate the burden of disease due to the impact of partial eradication and the inability to suppress or delay the relapses from the liver stage of *P. vivax*.

## Competing interests

The authors declare that they have no competing interests.

## Authors' contributions

MK, SY, RV, PMP, PS, PE, NMA, ET and RNP designed and supervised the study. LB, EK, NW, RM, KD, collected the data and carried out the preliminary analysis. MK, SY and RNP analysed the data and wrote the first draft of the manuscript. All authors contributed to drafting and approving the final manuscript.
